# Biomarkers of Osteosarcoma, Chondrosarcoma, and Ewing Sarcoma

**DOI:** 10.3389/fphar.2017.00150

**Published:** 2017-04-07

**Authors:** Francesco R. Evola, Luciano Costarella, Vito Pavone, Giuseppe Caff, Luca Cannavò, Andrea Sessa, Sergio Avondo, Giuseppe Sessa

**Affiliations:** Clinica Ortopedica, Dipartimento di Chirurgia, Azienda Ospedaliera-Universitaria Policlinico Vittorio Emanuele di CataniaCatania, Italy

**Keywords:** biomarkers, osteosarcoma, chondrosarcoma, Ewing sarcoma

## Abstract

Osteosarcoma is the most frequent malignant bone neoplasm, followed by chondrosarcoma and Ewing sarcoma. The diagnosis of bone neoplasms is generally made through histological evaluation of a biopsy. Clinical and radiological features are also important in aiding diagnosis and to complete the staging of bone cancer. In addition to these, there are several non-specific serological or specific molecular markers for bone neoplasms. In bone tumors, molecular markers increase the accuracy of the diagnosis and assist in subtyping bone tumors. Here, we review these markers and discuss their role in the diagnosis and prognosis of the three most frequent malignant bone neoplasms, namely osteosarcoma, chondrosarcoma, and Ewing sarcoma.

## Introduction

Neoplastic bone disease includes metastatic lesions and primary bone neoplasms. We divide primary bone neoplasms into two groups: benign and malignant bone neoplasms.

Primary malignant bone neoplasms are quite rare and account for 0.2% of all human neoplasms. The incidence of primary malignant bone sarcomas compared with that of soft tissue sarcomas shows that osseous neoplasms are approximately 1/10 of their soft tissue counterparts (Fletcher et al., 2002).

According to the SEER registry, from 2005 to 2009, on average, the diagnosis of cancer is made around 41 years. Approximately, 54.5% were diagnosed in under 44-year-olds (28.2% under 20) and 32.2% in over 55-year-olds. Annually, the incidence rate was 0.9 per 100,000. In the US, the median age at death for bone cancer is 58 years. Approximately, 28.3% died under 34 years. The mortality for year is 0.4 per 100,000 (patients who died in 2005–2009 in the US) (Howlader et al., 2002).

Osteosarcoma is the most frequent malignant bone neoplasm (36% of cases), followed by chondrosarcoma (20–25%) and Ewing sarcoma (16%).

The etiology of malignant bone cancers is largely unknown. Some precancerous conditions, e.g., Paget disease, radiation injury and benign cartilaginous dysplasia’s have been identified, and recognizable syndromes with a high risk of malignant degeneration have been described (Ollier disease, Maffucci syndrome), although the majority of primary bone cancers arise *de novo* (Fletcher et al., 2002).

The clinical presentation of bone tumors is non-specific. Pain, swelling and general discomfort are the most common symptoms. Pathologic fractures are often tardive manifestations of the disease. Other clinical features include: limitation of movement, hyperthermia of the skin, loss of weight and alteration of anatomic profile with a visible mass (Singer and David, 2008; Jawad and Scully, 2010; Szuhai et al., 2012).

The diagnosis of bone neoplasms is histological, through a biopsy. Clinical and radiological features are important to integrate diagnostic algorithms and to complete the staging of bone cancer. Tumor staging, for prognostic purposes, includes the degree of differentiation and the local and distant diffusion.

The biological classification allows to evaluate the behavior of the malignant lesion. There are different grading systems with two (TNM two grade system), three or four grades. The higher the grade, the more aggressive is the cancer. The classification of sarcomas is based on criteria of Broders (1920) for grading squamous cell carcinoma (Fletcher et al., 2002).

In order to stage a neoplasm, we consider the grading together with other parameters such as local extension, and lymphonodal and distant metastasis. There are different staging systems based on clinical, radiological and histological features.

The TNM staging system is also valid for bone neoplasm, but is not commonly used because sarcomas do not usually metastasize to lymph nodes.

The Enneking surgical staging system for malignant mesenchymal tumors takes into account the surgical grade (G, G1, G2), local extent (T, T1, T2), and presence or absence of metastasis (M0, M1).

The staging system for malignant musculoskeletal sarcomas consists of three stages. Stage III represents any tumor with distant metastasis. Stages I and II are based on surgical grade of the tumor. Each stage is further divided into two subcategories (A, B) based on the local extent of the tumor. This last parameter, i.e., localization, is not taken into account by the Enneking system and represents one of its limits.

While the role of radiology and histology in the diagnosis, prognosis and treatment of bone neoplasm has been well-defined for several years, the role of biochemical markers (serum, genetic or histological markers) is still unclear.

The purpose of this review is to focus on these markers and, based on the literature of the last 5 years, to discuss their real function in the diagnosis and prognosis of the three most frequent malignant bone neoplasms: osteosarcoma, chondrosarcoma, and Ewing sarcoma.

## Biomarkers

There are several biomarkers and these can be specific or aspecific; diagnostic, prognostic, or therapeutic; serological, genetic, or histological. In accord with Joerger M, Huober J, and extending their discussion of bone turnover markers to markers in general, “the use of markers in oncology includes monitoring treatment in patients with malignant disease (therapeutic monitoring), predicting the risk of bone relapse in patients with a first diagnosis of potentially curative, early-stage malignant tumors (prognostic use), and making an early diagnosis of (microscopic) malignant bone disease in patients with a known malignant tumor to start early bone-targeted treatment and avoid skeletal-related events (diagnostic use)” (Joerger and Huober, 2012).

### Serological (Bone Formation and Resorption) Biomarkers

This group includes a series of serological markers which reflect the osteoblastic and osteoclastic activity of the bone. They can have diagnostic and prognostic value and can be useful in monitoring therapies, although there are no guidelines recommending their use in clinical practice. Their levels can alter under different conditions, physiological or pathological, and thus they are not specific.

In cancer, Biochemical markers are useful to evaluate the dissemination a distance, the response to therapy and survival. Metastases can be classified, based on radiological features, as osteolytic, osteoblastic, or mixed. The osteoblastic lesions are manifested by high levels of bone turnover markers. High levels of these markers again predict poor prognosis (Singer and David, 2008).

The use of bone markers in malignant tumors allows an early diagnosis, fast start of therapy, limited complications and predict the risk of relapse in patients with early-stage malignant tumors (Joerger and Huober, 2012).

In patients with osteolytic lesions, Elevated levels of urinary NTx causes a greater risk of complications and progression of cancer (Singer and David, 2008).

There is limited evidence that bone turnover markers can be used to predict bone metastases in patients with early-stage malignant tumors, with PINP (N-terminal propeptide of type 1 procollagen), ICTP (carboxyterminal cross-linked telopeptide of type I collagen) and bone sialoprotein (BSP), with tumor immunoexpression of BSP being the most studied and discussed. In the diagnostic process, serum bone-specific alkaline phosphatise (BSAP), PINP and osteoprotegerin (OPG) have been shown to be associated with bone metastases of lung or breast. However, the sensitivity of these markers is very low, suggesting that they cannot be preferred over conventional bone scans in the diagnosis of bone metastases. Greater sensitivity in the diagnosis of bone metastases was found for urinary NTx (N-terminal cross-linked telopeptide of type I collagen) and serum ICTP in solid tumor patients, serum TRAcP-5b (tartrate-resistant acid phosphatase type 5b) in patients with breast cancer, and serum BSAP, PINP and OPG in prostate cancer patients (Singer and David, 2008; Joerger and Huober, 2012).

### Molecular Biomarkers

In bone tumors, molecular diagnostics can increase the accuracy of the diagnosis and assist in subtyping bone tumors.

In their review, Szuhai et al. (2012) divide the genetic changes found in sarcomas into three groups: sarcomas with specific translocations (Ewing sarcoma, aneurysmal bone cyst), tumors with gene mutations or amplifications (chondrosarcoma, fibrous dysplasia, Chordoma), and sarcomas with genetic instability.

Different techniques can be used to detect cytogenetic changes, such as banding and multicolor fluorescence *in situ* hybridization, array comparative genomic hybridization (array CGH), targeted detection techniques (FISH, RT-PCR) and mutation detection techniques. Taking Ewing sarcoma as an example, the EWS/FL1 fusion protein is a well-known oncogenic factor in Ewing sarcoma. Recently, Robin et al. (2012) showed that EWS/FLI1 mediates up regulation of EYA3. The reduction of EYA3 protein has negative effects on the survival of Ewing sarcoma. The authors thus proposed EYA3 as a novel mediator of chemoresistance in Ewing sarcoma (Robin et al., 2012).

Yang et al. (2012) recently provided another example of molecular targets for therapeutic use. The authors have shown that all-trans retinoic acid (ATRA) inhibits the differentiation and growth of human OS through the activation of SMAD, suggesting a possible role for retinoic acid, in addition to classic chemotherapy, in the therapy of OS (Yang et al., 2012).

Saini et al. (2012) through transcriptome, proteome and Immunophenotyping for cell-surface markers, discover a decrease of CD24, CD326 and CD44, and higher ABCG2 and CBX3 expression in osteosarcoma TSC enriched cultures respect to non-enriched samples. The authors thus identify ABCA5 as a putative biomarker of TSCs and/or osteosarcoma (Saini et al., 2012).

## Osteosarcoma

Osteosarcoma is the most prevalent malignant bone tumor (Meyers and Gorlick, 1997), accounting for 30–80% of primary skeletal sarcomas (Link and Eilber, 1989). The peak incidence is at age 10–30 years (Bielack and Bernstein, 2005). Osteosarcomas predominantly target the long cylindrical bones, including the knee joint (approximately half of observations) and the humerus (Mankin et al., 1996). Among the most affected are the femur, tibia, humerus and, less frequently, shoulder blade, and bones of the pelvis and skull (Enneking et al., 1980). Approximately 20% of patients present lung metastases at initial diagnosis and, additionally, in 40% of patients metastases occur at a later stage. Eighty percent of all metastases arise in the lungs, most commonly in the periphery of the lungs, and exhibit resistance to conventional chemotherapy (Kager et al., 2003; Harting and Blakely, 2006; Bacci et al., 2008; Bielack et al., 2008; Hughes, 2009; Messerschmitt et al., 2009; Posthuma DeBoer et al., 2011). The 5-year survival rate for OS patients with metastases is 20%, compared to 65% for patients with localized disease, and most deaths associated with OS are the result of metastatic disease (Bielack et al., 2002; Eccles and Welch, 2007; Rodriguez et al., 2008; Pradelli et al., 2009). Survival of patients has improved with the discovery of new chemotherapies (Chou and Gorlick, 2006; Longhi et al., 2006; Perbal et al., 2009; Savitskaya et al., 2012). The new biotechnological and pharmacological research is directed on the use of markers serum tumor for treatment of osteosarcoma. Several international study groups started a multicenter study on the tumor markers (Fuchs et al., 1998; Ham et al., 2000; Woodgate, 2000; Lewis and Nooij, 2003; Bielack et al., 2009; Franke et al., 2011).

### Serum Markers

Kaseta et al. (2007) analyzed the expression of genes bax, caspase-8 and cytochrome c in subjects with osteosarcoma. They performed an immunohistochemical analysis of 35 surgically treated patients with primary OS and 18 tissue specimens from non-malignant osseous lesions. The authors suggest that none of the genes has a predictive role on survival, but the decrease of 4-year disease-free survival in the group compared with the control group, confirmed that more intensive adjuvant treatment could reduce the recurrence rates of the disease (Kaseta et al., 2007).

Contrary to previous hypotheses regarding c-erbB-2 and its potential role as prognostic biological marker, the expression of c-erbB-2 isn’t associated with the risk of metastases (Yalçin et al., 2008).

Zhao et al. (2008) assess the distribution of ribonucleoprotein hnRNP A2/B1 in the nuclear matrix in human osteosarcoma MG-63 cells, saying that this nuclear matrix protein has an important role on regulation of cell differentiation.

Luo et al. (2008) study the role of osteoblast in the development of osteosarcoma.

The author affirms that levels of alkaline phosphatase (ALP), Runx2, OSX, and osteopontin (OPN) were low in OS lines, because most OS cells fail to complete terminal differentiation. Their results suggest that alterations in osteoprogenitors may disrupt the osteogenic differentiation pathway. Thus, identifying potential differentiation defects in OS tumors would make it possible to reconstruct the tumorigenic events in osteoprogenitors and to develop rational differentiation therapies for clinical OS management (Luo et al., 2008).

Based on the concept that glycoproteins and glycosaminoglycans are an integral part of bone and prolonged exposure to fluoride for long duration has been shown to cause degradation of collagen and ground substance in bones, Sandhu et al. (2011) analyzed serum fluoride, sialic acid, calcium, phosphorus, and ALP levels in patients with osteosarcoma. They found that, compared to control groups, serum sialic acid concentration was significantly increased in patients with osteosarcoma and, in a second study, in patients with other bone tumors (Sandhu et al., 2011).

Guo et al. (2010) assesses the role of heat shock protein gp96 (HSPgp96) in human osteosarcoma. HSPgp96, was mainly expressed in the cytoplasm of osteoblastic sarcoma with less differentiation. The authors affirm that the HSPgp96 has a role in the pathogenesis of bone tumor, but the marker cannot be used in determining the degree of malignancy and as a target for tumor immunity (Guo et al., 2010).

Babkina et al. (2009) compare serum levels of endostatin, placental growth factor (PlGF) and fibroblast growth factor-1 and -2 (FGF-1 and FGF-2) among patients of osteosarcoma and a group of control patients; the author found increased levels of PGF-2, PIGF and endostatin, while the levels of FGF-1 are increased of 2.5 times (*p* = 0.004) (Babkina et al., 2009).

Yu et al. (2010) compare the expression of mitotic arrest defective protein 2 (MAD2) in primary osteosarcoma and in a control group by immunohistochemistry; the author notes an increase of this protein in human osteosarcoma, especially in patients with metastasis and poor survival (Yu et al., 2010).

Zhang et al. (2010) used gas chromatography mass spectrometry approach and profiled small-molecule metabolites to observe metabolic variations in urine and serum of patients with osteosarcoma; the author notes a disrupted energy metabolism, down-regulated lipid metabolism, dysregulated sugar levels, up-regulated amino acid metabolism, increase of glutathione metabolism and polyamine metabolism (Zhang et al., 2010).

The expression and clinical significance of cysteine-rich protein with Kazal motifs (RECK) was immunohistochemically examined in 49 osteosarcoma patients by Xu et al. (2010). The study confirmed that reduced RECK expression is a significant factor of poor prognosis (*p* = 0.017). RECK status is a useful prognostic factor in osteosarcoma and an independent prognostic factor contributing to the determination of more adequate therapy strategies for individual patients (Xu et al., 2010).

Using RT-PCR and Western blot assays to detect IGF-1R mRNA and protein expression in 26 osteosarcoma and non-cancerous bone tissues, Wang et al. (2012) concluded that insulin-like growth factor-1 receptor (IGF-1R) is an independent prognostic marker for osteosarcoma patients and that an increased expression of this molecule is correlated with metastasis of osteosarcoma (Wang et al., 2012).

Observing that cysteine-rich intestinal protein 1 (CRIP1) has a significant prognostic impact in gastric cancer, Baumhoer et al. (2011) investigated its role in osteosarcoma. They analyzed 223 pre-therapeutic and well-characterized osteosarcoma samples for the immunohistochemical expression of CRIP1 and correlated their findings with clinic-pathological parameters including follow-up, systemic spread and response to chemotherapy. The expression of this factor is predominant in patients with longer survival without metastases (Baumhoer et al., 2011).

Shimizu et al. (2012) performed a study to test the role of the factors fibroblast growth factor-2 (Fgf2) or leukemia-inhibitory factor (Lif) in the maturation of osteosarcoma cells. These factors may reduce the osteogenic differentiation of osteosarcoma cells. Also Fgf2 helps the proliferation and migration of neoplastic cells, and alters the response of cells to drug therapy. The block of Fgf2 factor could modulate the progression of osteosarcoma (Shimizu et al., 2012).

Lu et al. (2012) assess the role of WNT-5a and ROR2 markers in development of various tumors through the expression of WNT. The authors, by immunohistochemistry, observed the expression of these two markers in patients with osteosarcoma and osteochondroma. The authors say that contemporary expression of both proteins is present in advanced stages and promotes the diffusion of the tumor; moreover, gender, age and the morphological variation do not have a significant role (Lu et al., 2012).

Nunez et al. (2012) observed the expression of CD 138 (syndecan-1), normally found in epithelial tumors and hematologic diseases, and affirm that the presence of this marker in tumor bone cells does not exclude with certainty plasmatic origin.

Jin et al. (2012) assess the role of gelsolin as a tumor marker and therapeutic target in osteosarcoma. They characterized the differential expression of protein biomarkers in osteosarcoma serum, finding 58 significant protein spot features in osteosarcoma sera. Further, Western blotting and enzyme linked immunosorbent assay (ELISA) confirmed decreased levels of gelsolin in the osteosarcoma serum samples (Jin et al., 2012).

Nestin protein has been detected in various malignancies and its expression correlates with advanced grade in some neoplasms. Zambo et al. (2012) examined its possible role in osteosarcoma. Using immunohistochemistry and immunofluorescence, they evaluated nestin expression in tumor tissue samples from 45 patients with high-grade osteosarcomas. Nestin-positive tumor cells were detected in all of the examined osteosarcomas. Using immunofluorescence, high levels of nestin expression were associated with poorer clinical outcomes. Despite the significantly shorter survival rates observed in patients with elevated levels of nestin expression, nestin does not appear to represent a powerful prognostic marker that would be superior to conventional methods (Zambo et al., 2012).

A study by Tan et al. (2012) evaluated preferentially expressed the antigen of melanoma (PRAME), normally present in several human cancers, assessing that this marker plays a role in the proliferation and spread of bone tumor, although the mechanisms are unknown. In addition, the author affirm that this marker is associated with a poor prognosis and spread in the lungs (Tan et al., 2012).

The chemokine, molecules involved in tumor genesis, have been studied by Li et al. (2011). The author observed an increase in serum levels in patients with osteosarcoma, particularly CXCL4 and CXCL, and affirmed that the marker play a role in clinical results and new studies are needed for the treatment (Li et al., 2011).

Sharili et al. (2011) investigated the role of biomarkers Snail2, commonly observed in skin tumors, in bone tumors. The author observed that the expression of this marker is correlated with the severity of the tumor and the risk of metastases. In addition, the biomarker Snail2 is useful in the prognosis of bone tumors (Sharili et al., 2011).

Interestingly, Funovics et al. (2011) assessed the pre-operative serum C-reactive protein levels (CRP) in 79 patients undergoing resection of an osteosarcoma. The author asserts that CRP is an marker for survival in patients with high-grade tumor, and invites further large-scale studies to confirm these results (Funovics et al., 2011).

### Genetic Markers

Perbal et al. (2008) evaluated the expression of CCN1 genes, CCN2, and CCN3 in osteosarcoma; the author finds a synchronous and ordered expression of these genes during osteoblast differentiation in patients with osteosarcoma. In addition, the author affirms that CCN1 and CCN2 genes haven’t a role in the prognosis. In contrast, assessment for CCN3 expression levels at diagnosis may represent a useful molecular tool for early identification of patients with different prognoses (Perbal et al., 2008).

Gillette et al. (2008) investigated a human osteosarcoma cell line (OS 99-1), demonstrating the potentiality of this cell line to form *in vitro* calcium nodules upon exposure to mineralization inducing conditions and to represent an osteoblastic rather than chondroblastic OS by displaying several osteoblast-specific markers including collagen I, BSP, OC and ALP, whereas chondroblastic markers (aggrecan and LINK) were undetectable. However, this cell line provides a useful tool for investigating the molecular mechanisms contributing to osteosarcoma and may have the potential to serve as a culture system for studies involving bone physiology (Gillette et al., 2008).

De Nigris et al. (2008) found that Y1 and VEGF/CXCR4 seem to intervene in the pathogenesis of the malignant phenotype of osteosarcoma by acting on cell invasiveness and metastasis growth, because the deletion of the gene produces a lower involvement of the cells by the tumor and a lower spread.

Zuffa et al. (2008) investigated the effect of TP53 (tumor protein 53, p53) on the genetic stability in patients with osteosarcoma. The author notes that the protein protects the DNA of cells, although are not known the mechanisms of protection exactly (Zuffa et al., 2008).

Wei et al. (2008) analyzed the c-kit gene in patients affected by osteosarcoma; the author notes that gene alteration of the protein is a prognostic marker of the tumor; moreover, exons 11 and 17 can’t be considered for the treatment of cancer through reduction of c-kit tyrosine kinase activity.

Nathan et al. (2009) observed, through a molecular analysis of cell line OS1, a genome instability after mutations that affect the function of the transcription factor Runx2. The author asserts that this cell line allows to identify possible molecular abnormalities that transform the osteoblast into cancer cells (Nathan et al., 2009).

The role of transcriptional regulators Oct-4 in osteosarcoma has been studied by Levings et al. (2009). According to the author this genetic marker plays a biological role in the proliferation and spread of cancer (Levings et al., 2009).

The expression of Mir-34s was reviewed by He et al. (2009) into two categories of osteosarcoma: U2OS (p53 +/+) and SAOS-2 (p53 -/-). The author states that the action of miR-34s is p53-dependent, and causes alteration of proliferation and apoptotic process. He et al. (2009) observed a reduced expression and inactivation of miR-34 gene in a group of osteosarcomas.

Yang J. et al. (2010) evaluated the role of the WWOX gene and, through comparative genomic hybridization, showed how the elimination or downregulation affects the survival of patients with osteosarcoma; also the author believes that the phenotypic alteration affects the early phases of the disease.

Analyzing blood samples of 168 osteosarcoma patients, Hu et al. (2010) used PCR amplification and DNA sequencing to determine the TGFBR1^∗^6A variant, a dominant polymorphism of the transforming growth factor β receptor 1 (TGFBR1). Their conclusion was that TGFBR1^∗^6A is associated with increased susceptibility to, and metastasis diffusion of, osteosarcoma (Hu et al., 2010).

Mirabello et al. (2010) report an association study of common single-nucleotide polymorphisms (SNPs) across 8q24 to explore the role this region may play in osteosarcoma risk. The 8q24 chromosomal region contains several loci that are associated with the risk of many different cancers. The study suggested that several SNPs in 8q24 may be associated with osteosarcoma, but the susceptibility observed was modest (Mirabello et al., 2010).

Bcl-xL, a member of Bcl-2 protein family functioning as dominant regulators of apoptotic cell death, has been reported to play important roles in malignant transformation and tumor development. Wang et al. (2010) studied the expression of Bcl-xL in osteosarcoma with therapeutic aims. The author, through many genetic techniques, compare the expression of this gene in tumor and non-tumor cells. An increase of Bcl-xL mRNA was observed in cancer cells with metastases compared to non-metastatic cells; also the expression of gene was significantly higher in tissues with tumor compared to healthy ones. Therefore, the author believes that the increase of the expression of Bcl-xL mRNA has a role in the diffusion of osteosarcoma and can be a useful molecular target for the treatment (Wang et al., 2010).

Based on many papers reporting that the reduction of expression of Fas protein is correlated to a higher risk of lung metastases and arsenic trioxide (ATO) may promote cell apoptosis in cancers. Yang G.F. et al. (2010) evaluated the role of ATO and the degree of expression of the Fas protein of human osteosarcoma cells (Saos-2 cell line); the author affirms that ATO reduces cell proliferation according to the dose and time and increased the expression of the Fas protein, although other mechanisms are interested in this process (Yang G.F. et al., 2010).

Wang et al. (2011), assert that the + 49G/A polymorphism of cytotoxic T-lymphocyte antigen-4 (CTLA-4), a molecule that decreases the immune response mediated by T-cells, promotes the development of osteosarcoma.

Lockwood et al. (2011) performed an analysis of DNA in osteosarcoma tumor samples. The overexpression of cyclin E1 was linked to potential prognostic and therapeutic implications (Lockwood et al., 2011).

**FIGURE 1:**
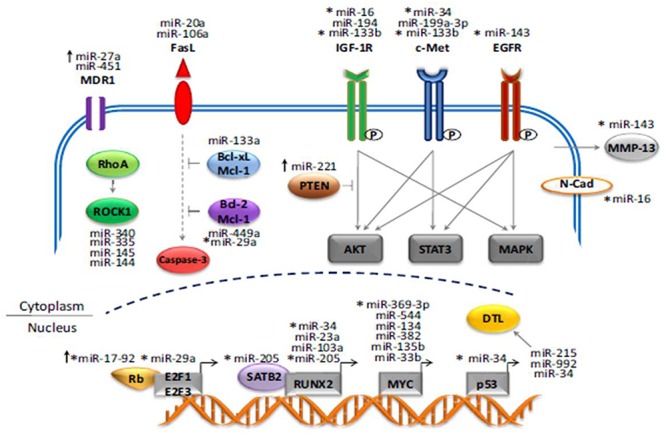
**MiRNA genes that play a role in the development and progression of Osteosarcoma**.

A study performed by the Massachusetts General Hospital group asserts that miR-199a-3p is involved in proliferative process of osteosarcoma. The restoration of this marker may provide therapeutic benefits in osteosarcoma. MicroRNAs (miRNA, miR) play an important role in cancer cell growth and migration. However, the potential roles of miRNAs in osteosarcoma remain largely uncharacterized. By applying a miRNA microarray platform and unsupervised hierarchical clustering analysis, they found that several miRNAs have altered expression levels in osteosarcoma cell lines and tumor tissues when compared with normal human osteoblasts. Three miRNAs, miR-199a-3p, miR-127-3p and miR-376c, were significantly decreased in osteosarcoma cell lines compared to osteoblasts, whereas miR-151-3p and miR-191 were increased in osteosarcoma cell lines (Duan et al., 2011) (**Figure 1**).

A preliminary study by Folio et al. (2011) supports the hypothesis that overexpression of Cortactin (CTTN) gene, contained in the 11q13 amplicon, is involved in osteosarcoma carcinogenesis. The potential of cortactin overexpression as a biomarker for osteosarcoma is, in fact, consolidated and transcriptomic profiling has shown cortactin to be overexpressed in pediatric osteosarcoma. The CTTN represents, according to the author, a valid biomarker for cancer (Folio et al., 2011).

Shen et al. (2012) examined the expression and localization of hArgBP2 in osteosarcoma MG-63 cells. After the successful construction of a recombinant plasmid of hArgBP2 gene, they identified the expression of GFP-hArgBP2 fusion mainly localized in the cytoplasm and perinucleus of MG-63 cells. The protein was identified and isolated using GFP antibody (Shen et al., 2012).

Yang et al. (2011), using microarray-based comparative genomic hybridization (aCGH), reported for the first time That vascular endothelial growth factor (VEGF) pathway genes, including the VEGFA protein, is overexpressed and play a role in poor prognosis and reduced survival in osteosarcoma (Yang et al., 2011).

Chen et al. (2012) used immunohistochemical staining in serial sections of the osteosarcoma analyzed to investigate expression patterns of IGF2 and IMP3 and their relationship with angiogenesis in the tumor. The author states that the increased expression of IGF2 and IMP3 can promote tumor angiogenetic processes (Chen et al., 2012).

### Prognostic Factors

Western blot analysis showed that expression of CRM1 (an intermediary involved in the transport of essential molecules of the cell, such as proteins and messenger RNA) was significantly increased in osteosarcoma compared with normal tissues (Yao et al., 2009).

Won et al. (2009) analyzed the role of Runx2 protein in bone tumor; the author affirms that an increase in its expression results in an higher risk of osteosarcoma spread, and therefore considers the protein a valid prognostic marker.

Genetic alterations of glutathione *S*-transferase supergene family can change the body’s defense mechanisms to carcinogens, increasing the risk of cancer and pharmacoresistance. Salinas-Souza et al. (2010) investigated the genotype frequencies of GSTM1, GSTT1 and GSTM3 genes in 80 osteosarcoma patients and 160 normal control participants, and also the influence of these polymorphisms on the clinical outcome of osteosarcoma patients. They concluded that GST polymorphisms may have a role in treatment response and osteosarcoma progression (Salinas-Souza et al., 2010).

## Ewing Sarcoma

Ewing’s sarcoma is one of the few solid tumors for which the underlying molecular genetic abnormality has been described: rearrangement of the EWS gene on chromosome 22q12 with an ETS gene family member. These translocations define Ewing’s sarcoma family tumors (ESFT) and provide a valuable tool for their accurate and unequivocal diagnosis. They also represent ideal targets for the development of tumor specific therapies.

### EWS-FLI Gene

Ewing’s sarcomas is a particular type of bone tumor due to translocations of genes with formation of EWS-ETS fusion proteins, in particular EWS-FLI, a transcription factor more involved in the neoplasm. Since the etiology of Ewing’s sarcomas is unknown, several models have been developed with the aim to identify EWS-FLI target genes.

Hancock and Lessnick (2008) through a meta-analysis have identified the genes that are altered by EWS-FLI. This study allowed us to confirm the involvement of known genes and the identification of new genes. The comparison of the data from this study and those published on mesenchymal stem cells has allowed to affirm that these cells are potential precursors of tumor (Hancock and Lessnick, 2008).

Potikyan et al. (2008) detect a fibroblast, called IMR-90, which, through genetic modification, expresses EWS/FLI1; the author found that these cells go to neoplastic degeneration following the expression of this transcription factor.

Through a genome-wide high-resolution analysis, Savola et al. (2009) identify genetic areas with more involvement by transcription factor EWS/FLI1. In most cases, the changes affect the chromosomes 1q, 2, 8,9p, 12, end 16q. The authors conclude that this method could be an effective way of identifying chromosome regions and new genes affected by cancer (Savola et al., 2009).

Bachmaier et al. (2009) notes that the alteration of glycosylation of EWS-FLI1 allows to modify his tumor action and, therefore, is a useful aspect for the pharmacological treatment; the author notes that the glycosylation and phosphorylation of EWS-FLI1 modify the molecular weight and then the functionality of the transcription factor. Through mutation analysis, O-GlcNAcylation was traced to Ser/Thr residues of the amino-terminal EWS transcriptional-activation domain. Metabolic inhibition of the hexosamine biosynthetic pathway abrogated O-GlcNAcylation of EWS-FLI1 and interfered specifically with transcriptional activation of the EWS-FLI1 target Id2 (Bachmaier et al., 2009).

Guillon et al. (2009) investigated EWS-FLI1-bound DNA sequences in two Ewing cell lines and showed that the transcription factor preferentially binds two types of sequences including consensus ETS motifs and microsatellite sequences. Moreover, in reporter gene experiments, the transcription activation is highly dependent on the number of repeats that are included in the construct. Importantly, *in vivo* EWS-FLI1-bound microsatellites are significantly associated with EWS-FLI1-driven gene activation. Together, these results indicate the likely contribution of microsatellite elements to long-distance transcription regulation and to oncogenesis (Guillon et al., 2009).

Szuhai et al. (2009) through the use of instruments to study the cellular genome, showed a translocation that is not known which affects both EWSRI and NFATc2. The involvement of NFATc2, a transcription factor, determines an abnormal immune response, secondary to altered function of T-cell (Szuhai et al., 2009).

Hypoxia is a major factor of cell regulation in bone tumors, through the activation of transcription factor “hypoxia-inducible factor-1” (HIF-1); Aryee et al. (2010) studied the effect of hypoxia on cell lines of ESFTs *in vitro*. The author affirms that the hypoxia allows to control EWS-FLI1 expression, resulting in variability of clinical and prognostic features in tumor (Aryee et al., 2010).

Vural et al. (2011) studied CD99 expression associated with EWS/FLI1 translocations in order to define the clinical and prognostic results of bone tumor. The author found a statistically significant relationship in CD99 expression in Ewing’s sarcoma, and therefore considers that this marker has a role in the diagnosis and prognosis of cancer (Vural et al., 2011).

The Rizzoli Experience, conducting a molecular diagnosis of EFT over 4 years (2006–2009), was reported in 2011. The authors study the genome of a group of patients with Ewing’s sarcoma. The authors find different types of genetic abnormalities as a result of translocation of chromosomes: five forms of EWSR1-FLI1, three forms of EWSR1-ERG, and one EWSR1-FEV (Gamberi et al., 2011).

**FIGURE 2:**
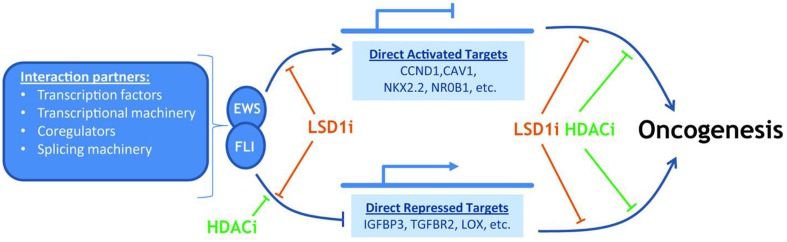
**EWS-FLI cause gene specific activation and repression**.

Erkizan et al. (2011) identified a potential scaffold for Ewing’s sarcoma, through the isolation of 27 binding peptides; the author found that the peptide ESAP1 shows a high binding to EWS-FLI1 in bone cancer. The minimal interaction region of ESAP1 was characterized and the lysine residues were found to be critical for cellular cytotoxicity. ESAP1 reduces the transcriptional activity of EWS-FLI1 and also disrupts cell cycle kinetics in Ewing tumor cells (Erkizan et al., 2011) (**Figure 2**).

### Other Genetic Markers

Douglas et al. (2008) investigated whether tumors of the Ewing sarcoma family express the BMI-1 gene, and whether it functions as an oncogene in this highly aggressive group of bone and soft tissue tumors. Their data showed that BMI-1 is highly expressed by ESFT cells and that, although it does not significantly affect proliferation or survival, BMI-1 actively promotes anchorage independent growth *in vitro* and tumorigenicity *in vivo*. The authors, moreover, found that BMI-1 promotes the tumorigenicity of both p16 wild-type and p16-null cell lines, demonstrating that the mechanism of BMI-1 oncogenic function in ESFT is, at least in part, independent of CDKN2A repression. The data suggests a pivotal role for BMI-1 in ESFT pathogenesis (Douglas et al., 2008).

A recent statistical methodology, called ICAN, allows to select genes that play a role in the prognosis and progression of tumors in studies with small samples, was reported by Bennani-Baiti et al. (2010) and tested in Ewing’s sarcoma (ES). Through this methodology the author affirms that CXCR4 expression increases the risk of tumor metastases, while CXCR7 expression is associated with shorter survival.

Some studies have shown that the EWS-ETS fusions, due to genetic changes, does not have a prognostic significance or Ewing’s sarcoma. Consequently, in an integrative genomic study of 105 ES tumors, Mackintosh et al. (2012) affirms the prognostic significance of 1QG and CDT2 expression in cancer, noting that this marker can be used in tumor therapy selectively (Mackintosh et al., 2012).

### Serum Markers

In Ewing’s sarcoma is present an increased expression of VEGF165 isoform; according to Huang et al. (2012) the reduced CAPER-α expression by EWS/FLI-1 causes an increased expression of this isoform.

An interesting study by the University of Texas group suggested that modulation of receptor activator NF-κ B (RANKL) by vascular endothelial growth factor-165 (VEGF165) can result in the activation of osteoclastic cells in Ewing’s tumor, through RANKL gene expression, and lead to the destruction bone. Based on the fact that VEGF165 induced osteoclast formation in murine bone marrow cells, the authors found that RANKL was unregulated in a dose- and time-dependent manner when ES cells were incubated with recombinant VEGF165 (Guan et al., 2009).

The role of the polycomb protein BMI-1 in Ewing’s sarcoma prognosis was studied by Cooper et al. (2011) the author has compared by genetic analysis two samples of bone tumors with an increased or decreased expression of this marker. They concluded that Ewing sarcoma family tumors (ESFT) which do not overexpress BMI-1 represent a novel subclass with a distinct molecular profile and altered activation of, and dependence on, cancer-associated biological pathways (Cooper et al., 2011).

FLI-1 has been described as a useful marker for Ewing sarcoma, particularly when hematolymphoid markers are negative. Using a monoclonal FLI-1 antibody, Lee et al. (2011) evaluated nuclear immunoreactivity in tumor cells in 10 small cell osteosarcomas, 10 mesenchymal chondrosarcomas, and 8 Ewing sarcomas, together with a number of other small, round, blue cell tumors. Their results showed that, in contrast to Ewing sarcoma, small cell osteosarcoma and mesenchymal chondrosarcoma lack FLI-1 immunoreactivity. FLI-1 is therefore useful in the differential diagnosis of small round blue cell tumors of the bone (Lee et al., 2011).

Considering Ewing sarcoma (ES) to be a systemic disease, Ash et al. (2011) used multiparameter flow cytometry (MPFC) to detect ES cells in the bone marrow (BM) of ES patients at diagnosis and to evaluate the prognostic significance of CD56 expression in BM samples. The authors observed that the cells had a contemporary expression of CD99-CD90-CD45, and therefore considered Ewing’s sarcoma a systemic disorder. Their conclusion was that CD56 expression could be used to identify ES patients (Ash et al., 2011).

Machado et al. (2011) assessed, by genetic analysis, the role of epithelial markers in Ewing’s tumor; the author to show the presence of epithelial markers used antibiotics and an immunohistochemical study in tumor cells. The authors say a statistically significant relationship between the expression of the markers and epithelial cell variants of the tumor is not present, even if they claim that an increased expression is present in these tumors and therefore can be regarded in the diagnosis of the disease (Machado et al., 2011).

Do et al. (2008) studied the correlation between HuR protein, a protein intended to stabilize the mRNAs, and Cyclooxygenase-2, an enzyme involved in the spread of tumors, in Ewing’s tumor; the author, who supposed that HuR can stabilize the mRNA of COX-2, does not observe a simultaneous increase of the expression of HuR protein and Cyclooxygenase-2 in cancer cells; also Do et al. (2008) believe that that relationship is indirectly evaluated by observing the mRNA stabilization.

## Chondrosarcoma

Chondrosarcomas are malignant cartilage tumors. The annual incidence is 1 in 1,000,000 people, with greater involvement of people with ages between 30 and 60; the disease affects more frequently the pelvis and lower limbs (Hogendoorn et al., 2010).

We can distinguish between primary and secondary chondrosarcomas. The chondrosarcoma may develop from other bone tumors such as osteochondroma and enchondroma. In these cases they can occur as multiple lesions.

There are four different histologic variants of chondrosarcoma (Fletcher and Unni, 2002):

(1) Mesenchymal chondrosarcoma is a highly malignant tumor, histologically similar to Ewing’s sarcoma, with foci of cartilaginous differentiation. This very aggressive type of chondrosarcoma has a high risk of local recurrence and distant metastasis.
(2) Dedifferentiated chondrosarcoma. These are aggressive neoplasms and have a poor prognosis.
(3) Clear cell chondrosarcomas are low-grade tumors. They typically involve the epiphyseal end of a long bone. Radiographs show a lytic defect at the epiphyseal end of long bones which is sharply demarcated with sclerotic margins. They carry a low recurrence rate and a good prognosis with wide resection.
(4) Extraskeletal myxoid chondrosarcoma, is a slow-growing tumor characterized, histologically, by prominent myxoid degeneration and a prolonged course, despite the high incidence of local recurrence (Yasuda et al., 2012). Extraskeletal myxoid chondrosarcoma has been considered by the World Health Organization as a differentiated tumors in cancer classification (Fletcher and Unni, 2002).

Chondrosarcomas can be classified into three histological grades.

Grade I (low grade) – hyperchromatic plump nuclei of uniform size and cytology very similar to enchondroma.
Grade II (intermediate grade) – Increased cellularity; hypercromasia; distinct nucleoli and foci of myxoid alteration.
Grade III (high grade) – with increased cellularity and nuclear atypia, occasional giant cells, abundant necrosis and presence of mitosis.

The higher grade tumors are the most malignant. Grade I lesions rarely metastasize, whereas 10–15% of grade II lesions and more than 50% grade III lesions metastasize.

Low-grade chondrosarcomas resemble benign cartilaginous tumors, and it is difficult to differentiate between the two lesions on the basis of histological features alone. Understanding the difference between a chondrosarcoma and its benign counterpart is crucial for the prognosis, as evidenced by several authors and by Weber in her case report (Weber and Raymond, 2002).

The role of biochemical and genetic markers in diagnosis, prognosis, and treatment of chondrosarcoma is unclear.

### Genetic Markers

Hallor et al. (2009) found a similar pattern of genomic imbalances in an high percentage (90%) of 67 chondrosarcoma cases. The author found deletions of loci at the level of CDKN2A, EXT1, and EXT2 genes, who are interested in the development of the disease. The alteration of specific gene regions suggests a potential role for genetic markers in diagnosis and prognosis of chondrosarcomas (Hallor et al., 2009).

Szuhai et al. (2012) reported that most peripheral chondrosarcomas had a higher proliferation rate on Ki-67 immunohistochemistry and that they were associated with loss of heterozygosity at many loci.

According to the ESMO/EUROBONET Working Group, the overexpression of p53 protein, 17p1 alterations, and TP53 mutations in high-grade chondrosarcomas suggest that the p53 mutation is a late event involved in tumor progression. Amplification of 12q13 and loss of 9p21 are genetic aberrations found in conventional chondrosarcomas. The loss of INK4A/p16 expression was shown to be restricted to high-grade chondrosarcomas, suggesting it plays a role in tumor progression (Hogendoorn et al., 2010).

Rozeman et al. (2005) showed that higher expression of PTHR1 and Bcl-2 was associated with increasing histological grade in chondrosarcoma, suggesting its involvement in tumor progression. Instead, PTHrP signaling is not important in the malignant transformation of enchondroma (Rozeman et al., 2005).

A recent study by Liang et al. (2012) investigated overexpression of Aurora Kinase A and B in chondrosarcoma and their relevance to prognosis. The expression of Aurora Kinase A and B was significantly higher in chondrosarcoma than in chondroma (*p* < 0.01). The author evaluates the expression of Aurora Kinase A and B in patients with recurrence and metastasis, and found that the marker expression is significant in these patients than in the control group (*p* > 0.05). Also observes that the expression of the marker is lower in low-grade tumors compared to medium and high grade tumor (*p* < 0.01). The sex and age does not show a statistically significant correlation with tumor prognosis. Liang et al. (2012) assert that the expression of Aurora Kinase A and B influences patient survival significantly. However the use of these markers as prognostic factors needs further study. They also propose Aurora Kinase A and B as therapeutic targets (Liang et al., 2012).

Amary et al. (2011) identified somatic heterozygous isocitratedehydrogenase 1 (IDH1) hot spots (R132C and R132H) or IDH2 (R172S) as mutations present in cartilaginous tumors but not in other mesenchymal tumors.

In their review, Szuhai et al. (2012) identified several active signaling pathways for central chondrosarcoma, including pRB (61,62), IHH/PTHLH/Bcl-2 (63e66), Src, Akt, and PDGFR with no effect of imatinib, IGF, or estrogen signaling, together with hypoxic and glycolytic pathways and the overexpression of the Bcl-2 family.

Signaling in dedifferentiated chondrosarcomas was also studied by Rozeman et al. (2009) in a case series of 16 dedifferentiated chondrosarcomas. The author compares peripheral chondrosarcomas with secondary peripheral chondrosarcomas and notes the lack of CD44v3 and lower BCL-2 expression. Authors found improved survival in peripheral dedifferentiated chondrosarcomas with PAI-1 expression. They concluded that in dedifferentiated peripheral chondrosarcomas PAI-1 might also be of interest as a prognostic marker both in peripheral and in central dedifferentiated chondrosarcoma (Rozeman et al., 2009).

Various authors have studied the role of extracellular receptor kinase proteins (ERK) in chondrosarcoma. Chandhanarat et al. (2012) analyzed the role of MAPK/ERK signaling in osteosarcoma, Ewing sarcoma and chondrosarcoma and affirms that there is no data on the effectiveness of the MAPK/ERK therapy for chondrosarcomas, although there are studies in the literature that show positive results in the treatment of metastatic osteosarcomas (Chandhanarat et al., 2012).

Sun et al. (2010) report that chondrosarcoma cell invasion is increased by hypoxia, induced CXCR4 and MMP1 expression, and is mediated by HIF-1a and ERK. With CXCR4 blocking, both invasion and MMP1 can be inhibited. Thus, the authors suggest the CXCR4/SDF1 signaling as a therapeutic target for chondrosarcoma (Sun et al., 2010).

In 2009, the same authors found that reduced expression of HDAC4 in chondrosarcoma cells increases expression of Runx2, leading to increased VEGF expression and *in vitro* angiogenesis. Thus, both hypoxia and dysregulated expression of a developmental pathway are causes of increased VEGF expression in chondrosarcoma (Sun et al., 2010).

Pedrini et al. (2011) analyzed the role of EXT mutations in 529 patient with multiple hereditary exostoses. Malignant transformation was observed in 5% of patients, and no evidence of association between chondrosarcoma onset and EXT mutation, sex, severity of disease or number of lesions was detected (Pedrini et al., 2011).

Tirino et al. (2011) states that CD133 marker is useful in the isolation of stem cells in bone tumors.

Zenmyo et al. (2010) studied the expression of GADD45b in chondrosarcoma and its role in tumor progression. The author says that GADD45b expression is inversely proportional to the histological grade of the bone tumor, and therefore, this marker is useful to study the degree of differentiation (Zenmyo et al., 2010).

### Biological and Molecular Markers

Although chondrosarcomas are considered to be chemo- and radio-resistant, Jamil et al. (2010) reviewed and proposed different molecular and genetic therapeutic targets for chondrosarcoma. The author states that methoxyestradiol reduces angiogenesis and cell proliferation, and therefore, this marker may lead apoptosis in chondrosarcoma’s cells and arrest the progression of the disease. PEDF has been shown to increase cellular apoptosis in chondrosarcoma. Jamil notes that Epigallocatechin-3-gallate has an anti-inflammatory action and positive effects in the treatment of chondrosarcoma. The author assesses the role of peroxisome proliferator-activated receptor gamma, a nuclear receptor that regulates the proliferation and apoptosis in different cancers; according to Jamil et al. (2010) this marker is present in chondrosarcoma and therefore can be used in the identification of illness.

Meijer et al. (2011) evaluated the role of aromatase and estrogen receptor alpha in bone tumor proliferation; the authors suggest that the presence of both markers in tumor cells in patients with chondrosarcoma indicates a higher risk of cell proliferation. But *in vitro* and pilot *in vivo* studies have shown no effect of estrogen-signaling inhibition on tumor growth. Thus, ESR1 could be a diagnostic or a prognostic marker but has little role in therapy (Meijer et al., 2011).

In recent years, various authors have examined the role of COX-2 expression in chondrosarcoma with its implications for therapy and, in particular, for prognosis. Schrage et al. (2010) analyzed COX-2 expression in cartilaginous tumors. They confirmed the expression of COX-2 in 65% of chondrosarcomas. They also found high COX-2 protein expression was mainly found in solitary peripheral chondrosarcoma and in enchondromatosis-related central chondrosarcoma, which was confirmed by qPCR. They emphasize the potential role of celecoxib in treatment (Schrage et al., 2010).

Nakagawa et al. (2010) reported that there was a significant association of nitrotyrosine, COX-2 and CD34 with histological grades of chondrosarcoma, suggesting that these markers have a role in patient survival.

Cintra et al. (2011) investigated the role of COX-2 expression and angiogenetic factors in chondrosarcoma progression. The authors observed that a high COX-2 expression adversely affects patient survival and a positive correlation between CD34 and COX-2 expression, which confirms the relationship between COX-2 and angiogenesis, suggesting a possible role for this marker in patient prognosis (Cintra et al., 2011).

Ariizumi et al. (2010) investigated the role of lymphatic marker podoplanin in chondrosarcoma. In their series, podoplanin was expressed strongly (10/10) by chondrosarcomas. Therefore, they concluded, this marker is useful for identification bone cancer (Ariizumi et al., 2010).

Papachristou et al. (2008) studied the role of Integrin-linked kinase, α and β-parvin, Mig-2 in chondrosarcoma. These molecules allow cell attachment to the matrix, motility and cell growth. According to the author these biomarkers are particularly expressed in this bone cancer and thus play a role in the progression and prognosis of the disease (Papachristou et al., 2008).

## Conclusion

During growth the most prevalent kind of tumor are osteosarcoma, chondrosarcoma, and sarcoma of Ewing. Current knowledge of the molecular biomarker involved in each type of tumor has led to better approaches in the treatment. Molecular markers increase the accuracy of the diagnosis and assist in subtyping bone tumors, thus improving the quality of life of patients. This review had the objective to summarize the biomarkers are more interested in these three types of bone tumors.

## Future Perspectives

Despite the large progress in the field, as the discovery of new markers (sCD30 and sCD40L), many challenges are still ahead. The search for biomarkers for non-invasive diagnosis of bone tumors is currently an area research interests. One of the most important aspects will be the identification of microRNAs involved in bone tumors. MicroRNAs are a group of small non-coding RNAs circulating in blood of patients, that play a role in post-transcriptional gene expression in normal and disease physiologies, even if its molecular mechanisms still remain elusive. MicroRNAs have been considered potential biomarkers and can be used for diagnosis, prognosis, and targeted treatment of neoplastic bone disease.

The differential expression profiles of miRNAs can be used as promising diagnostic and prognostic biomarkers of osteosarcoma, chondrosarcoma, and Ewing sarcoma; miRNAs play a positive role in the progression of bone tumors by regulating proliferation, invasion, metastasis, apoptosis and angiogenesis. The identification of more specific non-invasive biomarkers could help the treatment of bone tumors in the future.

## Author Contributions

All authors contributed to the drafting work and accepted the publication, in particular FE, LCo, and VP treated the molecular aspect, GC, LCa, and AS treated literature research, SA and GS revised the manuscript.

## Conflict of Interest Statement

The authors declare that the research was conducted in the absence of any commercial or financial relationships that could be construed as a potential conflict of interest.
